# Bacterial community structure and soil properties of a subarctic tundra soil in Council, Alaska

**DOI:** 10.1111/1574-6941.12362

**Published:** 2014-08-04

**Authors:** Hye Min Kim, Ji Young Jung, Etienne Yergeau, Chung Yeon Hwang, Larry Hinzman, Sungjin Nam, Soon Gyu Hong, Ok-Sun Kim, Jongsik Chun, Yoo Kyung Lee

**Affiliations:** 1 Korea Polar Research Institute, KIOSTIncheon, Korea; 2 School of Biological Sciences, Seoul National UniversitySeoul, Korea; 3 National Research Council of CanadaMontreal, QC, Canada; 4 International Arctic Research Center, University of Alaska FairbanksFairbanks, AK, USA

**Keywords:** soil bacteria, soil depth, pH, Alaska, pyrosequencing, tussock tundra

## Abstract

The subarctic region is highly responsive and vulnerable to climate change. Understanding the structure of subarctic soil microbial communities is essential for predicting the response of the subarctic soil environment to climate change. To determine the composition of the bacterial community and its relationship with soil properties, we investigated the bacterial community structure and properties of surface soil from the moist acidic tussock tundra in Council, Alaska. We collected 70 soil samples with 25-m intervals between sampling points from 0–10 cm to 10–20 cm depths. The bacterial community was analyzed by pyrosequencing of 16S rRNA genes, and the following soil properties were analyzed: soil moisture content (MC), pH, total carbon (TC), total nitrogen (TN), and inorganic nitrogen (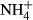 and 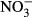). The community compositions of the two different depths showed that *Alphaproteobacteria* decreased with soil depth. Among the soil properties measured, soil pH was the most significant factor correlating with bacterial community in both upper and lower-layer soils. Bacterial community similarity based on jackknifed unweighted unifrac distance showed greater similarity across horizontal layers than through the vertical depth. This study showed that soil depth and pH were the most important soil properties determining bacterial community structure of the subarctic tundra soil in Council, Alaska.

## Introduction

The Arctic region is currently receiving much attention because global warming is predicted to be the greatest and most rapid at high latitudes (IPCC, 2007). Evidence collected from the past few decades indicates that warming is already underway in the Arctic (ACIA, 2005; Chapin *et al*., 2005). Arctic permafrost soils contain a significant amount of soil carbon (Schlesinger, 1997; Ping *et al*., 2008; Tarnocai *et al*., 2009), and the warming effect is making soil organic carbon more vulnerable (Grosse *et al*., 2011). A warmer climate will cause carbon stored in the soil to be released into the atmosphere via microbial decomposition (Bardgett *et al*., 2008; Schuur *et al*., 2009). According to current climate change projections, studying microbial processes in the subarctic region is important because the area is highly responsive and vulnerable to climate change (Christensen *et al*., 1998; Anisimov & Fitzharris, 2001).

Understanding the soil microbial community structure is essential to elucidate microbial processes. The bacterial communities have been characterized in various arctic soil environments including subarctic regions using culture-independent techniques, such as DGGE, T-RFLP, clone libraries, and next-generation sequencing (Männistö *et al*., 2007; Steven *et al*., 2007; Wallenstein *et al*., 2007; Lauber *et al*., 2009; Margesin *et al*., 2009; Campbell *et al*., 2010; Chu *et al*., 2010; Larose *et al*., 2010; Schütte *et al*., 2010; Yergeau *et al*., 2010; Coolen *et al*., 2011). In general, these investigations have shown that bacterial communities in the Arctic are similar in structure and diversity to bacterial communities of other biomes at the phylum level (Chu *et al*., 2010). However, few studies have examined the Arctic bacterial community structure at a lower taxonomic level (Männistö *et al*., 2007; Steven *et al*., 2007; Campbell *et al*., 2010; Larose *et al*., 2010), and such studies may reveal important differences in the actual functional groups of bacteria present in the Arctic.

Several reports have shown the relationship between bacterial communities and various environmental factors. For example, bacterial community composition is related to vegetation type, the quality of soil organic matter, geographical region, and environmental factors such as temperature, water, nutrient availability, soil pH, etc. (Fierer & Jackson, 2006; Wallenstein *et al*., 2007; Coolen *et al*., 2011). Currently, soil pH is considered as the most important factor influencing microbial community structure (Lauber *et al*., 2009; Chu *et al*., 2010; Shen *et al*., 2013; Bartram *et al*., 2014).

In the present study, we investigated the bacterial community structure and compositional patterns in subarctic tundra soils located in Council, Alaska, and explored the relationships between bacterial community and soil properties. For this investigation, we obtained a large amount of bacterial sequence data from soil samples through pyrosequencing to examine the bacterial community at a deep phylogenetic level. We then compared the bacteria present in the soil samples to determine which taxa were dominant in the community and how the bacterial community structure changed with soil properties.

## Materials and methods

### Ethics statement

Access for the study site was approved by the International Arctic Research Center (IARC) of the University of Alaska Fairbanks (UAF).

### Site description and sampling design

The study site is located in Council, on the Seward Peninsular in Northwest Alaska (64°51′N, 163°39′W), which is a subarctic region. The site is *c*. 30 m above sea level, and the annual mean air temperature and precipitation are −3.1 ± 1.4 °C and 258 mm, respectively (climate data were obtained from the International Arctic Research Center of the University of Alaska, Fairbanks). At the time of sampling (mid-August 2011), the depth of the active layer was *c*. 50–70 cm. The sampling site is composed of moist acidic tussock tundra, and the dominant vegetation was cotton grass (*Eriophorum vaginatum*) or tussock, blueberry (*Vaccinium uliginosum*), and lichen and moss (*Sphagnum* spp.) beds.

Thirty-six sampling points were selected over an area of *c*. 300 × 50 m. The points were spaced at 25-m intervals, resulting in a latticework of 12 points × 3 points. Before acquiring soil samples, we removed the aboveground vegetation and litter layer, and cleaned the shovel with 70% ethanol to prevent contamination between samples. At each site, soil samples were collected from a depth of 0–10 cm (upper-layer soil) and a depth of 10–20 cm (lower-layer soil). The collected soil samples were wrapped in autoclaved foil, placed in ziplock bags, and transported to the laboratory in a frozen state. The soils in sites 4 and 35 were saturated with water, and only the upper-layer soil was collected. Overall, 70 soil samples were collected for this study. The collected soil samples were stored at −20 °C until DNA extraction and soil analysis.

### Physical and chemical properties of soil

Gravimetric MC was determined by measuring the difference in weight between the field-moist soil samples and the same soil samples dried at 105 °C for 48 h. For inorganic nitrogen (N) analyses, *c*. 7 g of fresh soil was immediately set aside after sampling and kept frozen until extraction. Inorganic N (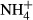 and 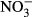) was extracted using a 2 M KCl solution and filtered through Whatman #42 paper. The filtrate was analyzed with an Auto-analyzer (Quaatro; Seal Analytical, Inc.). The remaining soil was air-dried and sieved through 2 mm mesh for further analyses. Soil pH was determined in a 1 : 10 soil : water (w/v) solution (Thomas, 1996). Soil was ground and passed through a 53 μm sieve to determine TC and TN content. TC and TN contents were measured by combustion (950 °C; FlashEA 1112; Thermo Fisher Scientific). Total inorganic carbon was negligible in all soil samples.

### PCR amplification and pyrosequencing

To extract genomic DNA from the soil samples, the soils were subsampled from the bulk soil, freeze-dried, and homogenized. Genomic DNA was extracted from 0.5 g of the homogenized soil samples using a FastDNA® SPIN kit for soil (MP Biomedicals) and a QuickPrep adapter (MP Biomedicals), according to the manufacturer's recommended protocol. The total DNA was quantified by Hoechst dye 33258 staining using a spectrophotometer with excitation and emission at 350 and 460 nm, respectively (Wallac EnVision 2013 Multilabel Reader, Perkin Elmer). The extracted DNA was stored at −20 °C until further analysis.

Genomic DNA extracted from the soil was amplified by PCR using the adapter-multiplex identifier–primer combinations targeting the V1–V3 regions (27F–518R) of bacterial 16S rRNA gene (Supporting Information, Table S4). The PCR reaction mixture (50 μL) contained 25 μL of master mix (DreamTaq™ Green PCR Master Mix [2×]), 1.4 μL of the forward and reverse primers (20 pmol of each primer), 1 μL of template DNA (1 ng μL^−1^), and 22.6 μL of deionized distilled water (DDW). The PCR program was as follows: an initial denaturation step at 95 °C for 3 min followed by 30 cycles of denaturation at 95 °C for 30 s, annealing at 56 °C for 30 s, and extension at 72 °C for 90 s, with a final extension at 72 °C for 7 min. All samples were amplified in triplicate, pooled in equal amounts, and purified using the QIAquick PCR Purification Kit (Qiagen). The PCR products were quantified with a NanoDrop. DNA sequencing was performed by DNALink (Seoul, Korea) using a GS-FLX 454 pyrosequencer (Roche).

### Processing of pyrosequencing data

PCR amplicon pyrosequencing data were processed using the qiime software package, ver. 1.7 (Caporaso *et al*., 2010a). Briefly, raw flowgrams (sff files) were filtered and noise and chimeras were removed using ampliconnoise software, ver. 1.27 (Quince *et al*., 2011), using the platform option for FLX Titanium sequence data implemented in qiime. Sequences with an average (± SD) length of 378 ± 45 bp were clustered based on operational taxonomic units (OTUs) at 97% similarity using uclust (Edgar, 2010). OTUs were assigned to taxa using the RDP Classifier method (Wang *et al*., 2007) with a training set based on the Greengenes database (release 13.5; Werner *et al*., 2011). Sequence alignments for phylogenetic reconstruction were generated using pynast software (Caporaso *et al*., 2010b) and the Greengenes database (DeSantis *et al*., 2006). Using additional downstream tools in qiime, a phylogenetic tree was built from the aligned sequences using fasttree 2.1 (Price *et al*., 2010), and a pairwise beta-diversity distance matrix for a randomly selected subset of 700 sequences was generated for all samples based on the unweighted unifrac phylogenetic distance metric (Lozupone *et al*., 2006).

To facilitate diversity comparisons among bacterial communities, we estimated diversity indices, including the Chao1, Shannon, and Simpson indices, for a randomly selected subset of 700 sequences from each sample to avoid effects of different sample sizes (Kirchman *et al*., 2010).

The 454 FLX Titanium flowgrams have been deposited in the National Center for Biotechnology Information (NCBI) Sequence Read Archive database (accession number, SRP026166).

### Statistical analysis

Statistical analyses were performed using r (version 3.0.0; The R Foundation for Statistical Computing) and primer-e v6 (Clarke & Gorley, 2006). Analysis of similarity (anosim) with 999 permutations and nonmetric multidimensional scaling (nmds) were conducted to compare bacterial community structure. Classification and regression tree (cart) analyses were conducted using rpart in the r software package (CP value set at 0.001) to determine which environmental variables explained the deviance of the dominant bacterial groups. A Mantel test was used to determine which physical and chemical properties of soil were significantly correlated with the bacterial community. To assess how the bacterial community changed with sampling distance (*c*. 22–427 m), a distance-decay relationship analysis, which assumes that community similarity will decrease with increasing geographical distance, was performed, as described in Martiny *et al*. (2011) with some modification. Briefly, the rate of distance-decay of the bacterial communities was calculated as the slope of a linear least squares regression on the relationship between geographic distance (m) versus the jackknifed unweighted unifrac distance of bacterial similarity, which is a qualitative metric of beta-diversity and is unaffected by the presence of duplicate sequences.

## Results

### Physical and chemical properties of soil

The soil at the study site was acidic and moist. The soil pH ranged from 3.90 to 5.02 (Table 1, Table S1). The upper-layer soil pH was slightly more acidic than the lower-layer soil pH (Table 1). Gravimetric MC was > 100%, except at site 17. The upper-layer soil contained higher MC than the lower-layer soil at most sites. In the upper-layer soil, the average TC and TN contents were 40% and 1.5%, respectively. The lower-layer soil had a lower TC content (36%), but the same TN content as the upper-layer soil. Therefore, the carbon and nitrogen (C/N) ratio was higher in the upper-layer soil. The ammonium (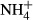) concentration was higher than the nitrate (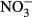) concentration at both depths and at all sites. Ammonium concentrations ranged from 8.6 to 93.1 μg g^−1^ soil, but the nitrate concentrations were negligible.

**Table 1:** The summary of physical and chemical properties of the subarctic tundra soil samples

	pH		TC (%)		TN (%)		C/N		MC (%)		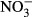 (μg N g^−1^ soil)		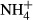 (μg N g^−1^ soil)	
							
	Upper	Lower	Upper	Lower	Upper	Lower	Upper	Lower	Upper	Lower	Upper	Lower	Upper	Lower
Mean	4.35	4.53*	39.94	35.94*	1.50	1.54	28.52	24.15*	628.2	438.4*	0.80	0.75	32.53	29.06
SD	0.29	0.28	6.81	12.35	0.46	0.58	9.05	6.56	222.5	252.6	0.48	0.53	22.25	17.91
CV (%)	6.61	6.25	17.04	34.36	30.38	37.52	31.71	27.14	35.4	57.6	59.18	70.56	68.40	61.61
MAX	5.02	5.01	43.87	48.55	2.23	2.38	66.30	56.93	1070.3	1201.6	3.19	3.29	93.08	91.55
MIN	3.90	3.96	2.10	1.85	0.09	0.08	19.31	18.26	53.4	32.1	0.27	0.21	8.62	9.79

TC, total carbon; TN, total nitrogen; C/N, a ratio of carbon to nitrogen; MC, moisture content; SD, standard deviation; CV, coefficient of variation.

* Denotes significant differences (*P* < 0.05) of soil properties between the upper and lower-layers.

**Fig. 1:**
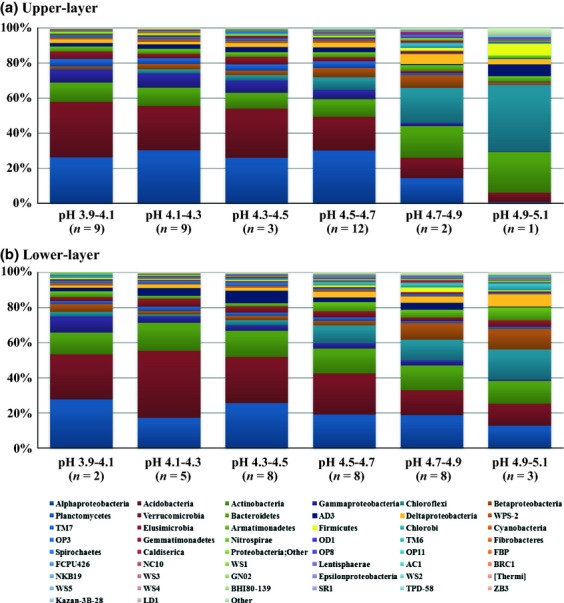
Relative abundance of phyla in the soil bacterial communities in the upper (a) and the lower-layer soils (b) separated according to pH.

The soil properties of site 17 were completely different from those of the other sites (Fig. 1). This site contained lower MC, TC, and TN contents, and higher soil pH because it was primarily composed of mineral layers rather than organic layers, which comprised the other sampling sites.

### General description of sequencing results

We obtained 91 742 good quality 16S rRNA gene sequences (V1–V3 region) from all the soil samples. On average, we obtained 1311 sequences (range, 718–1944 sequences) per sample. When we compared the soil bacterial communities using the same number of reads (700 sequences per sample), bacterial abundance and bacterial diversity were significantly higher in the upper-layer soils, as indicated by the Chao1 (*P* < 0.001), Shannon (*P* < 0.001), and Simpson (*P* < 0.05) indices (Table S2). OTUs accounted for 25.1–43.7% of the diversity, according to the Chao1 index (data not shown).

### Bacterial community structure within and between sites

The classifiable sequences comprised members of 50 phyla, including candidate phyla. The dominant phyla were *Acidobacteria*, *Alphaproteobacteria*, and *Actinobacteria*, which accounted for more than 40% of the bacterial sequences in all soil samples (Fig. 1). In addition, sequences of *Betaproteobacteria*, *Gammaproteobacteria*, *Chloroflexi*, *Deltaproteobacteria*, *Bacteroidetes*, *Verrucomicrobia*, *Planctomycetes*, *Chlorobi*, *Firmicutes*, *Elusimicrobia*, *Nitrospira*, *Armatimonadetes* (former candidate division OP10), *Gemmatimonadetes*, *Cyanobacteria*, *Spirochetes*, *Fibrobacteres*, *Caldiserica* (former candidate division OP5), *Lentisphaerae*, and *Epsilonproteobacteria* were also identified at relatively low abundances, as well as members of 27 candidate phyla and several unclassified bacteria (Fig. 1).

**Fig. 2:**
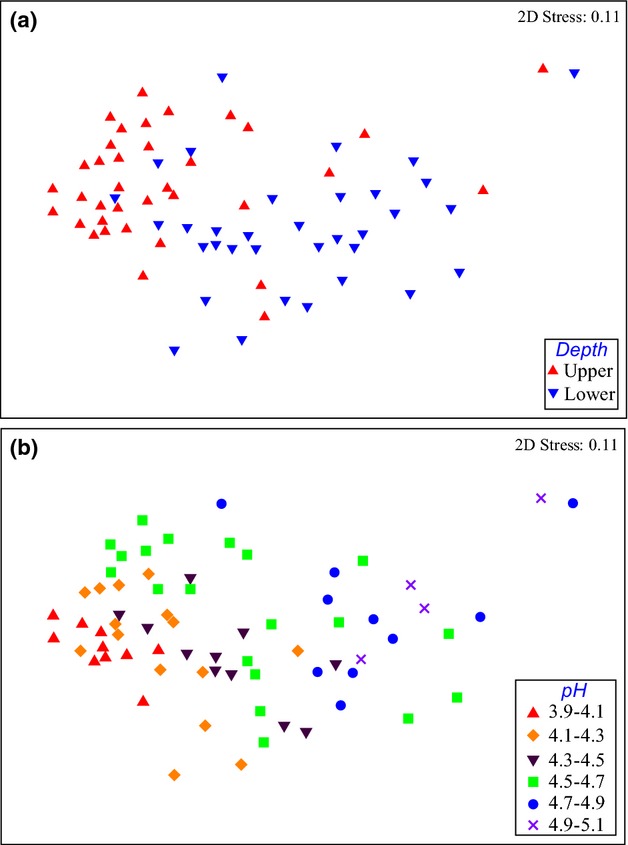
nmds plots derived from phylogenetic similarity based on jackknifed unweighted unifrac distances between soil samples, with symbols coded by depth (a) and pH (b).

**Fig. 3:**
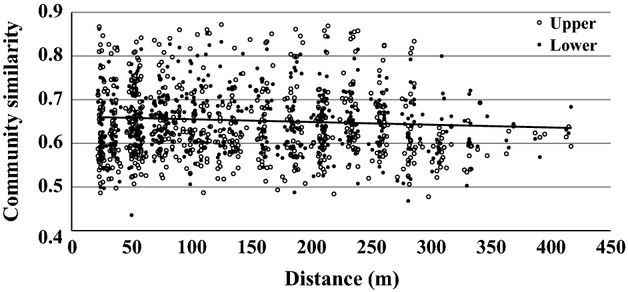
Distance-decay analysis of the relationship between geographic distance and bacterial community distance based on jackknifed unweighted unifrac distance in both layers. The slope was significant in the lower-layer soils (*P <* 0.05; *y* = −6*E*−05*x* + 0.6615, solid line).

In general, the bacterial community structures in the upper and lower-layer soils were different. *Alphaproteobacteria*, *Gammaproteobacteria*, and *Planctomycetes* were more abundant in the upper-layer soils, whereas *Actinobacteria*, *Betaproteobacteria*, *Chloroflexi*, and AD3 were more abundant in the lower-layer soils (Fig. S2). At the family level, *Methylocystaceae*, *Acetobacteraceae*, *Sinobacteraceae*, and Ellin6513 were more abundant in the upper-layer soil, whereas *Gallionellaceae*, *Solibacteraceae*, *Intrasporangiaceae*, and Ellin6529 were more abundant in the lower-layer soil (Fig. S2). The 16S rRNA gene sequences of the dominant OTUs, which accounted for more than 1% of the total sequences, were identified (Table 2). Only one OTU (OTU_1) showed > 97% sequence similarity with cultured bacteria, and most of the dominant OTUs have yet to be cultured (Table 2). The dominant bacterial OTUs accounted for 9.7% and 15.0% of the total sequences in the upper and lower-layer soil samples, respectively. Among the 11 dominant OTUs, three accounted for over 1% of total sequence in both soil layers. The nonmetric multidimensional scaling (nmds) plots indicated that the bacterial communities showed greater similarity across horizontal layers than through vertical depth (Fig. 2a). This pattern was confirmed by a significant anosim value (*r* = 0.338, *P* < 0.001) between the two depths. Bacterial communities were similar between sampling sites (Fig. 3). There was significant correlation between bacterial community similarity and sampling distance in the lower-layer soils (*P* < 0.05; Fig. 3); however, the relationship was not observed in upper-layer soils (*P* > 0.05).

**Table 2:** A list of dominant OTUs which were accounted for over 1% among total reads through eztaxon-e* database

					Relative abundance (%)	
					
OTU no.	The closest species (accession no.)	Detection source†	Pairwise similarity (%)	Lineage	Upper	Lower
1	*Afipia broomeae* (KB375282)	Human	99.5	*Alphaproteobacteria*	3.61	2.38
2	*Pseudolabrys* sp. (EU937836)	Biofilm	98.5	*Alphaproteobacteria*	1.44	2.91
3	*Telmatobacter* sp. (AJ292586)	Polychlorinated biphenyl-polluted soil	98.8	*Acidobacteria*	1.38	1.87
4	EU150278_s in *Steroidobacter*_f (EU150278)	Soil	100	*Gammaproteobacteria*	2.04	0.62
5	*Koribacter* sp. (AY913298)	Forest	98.8	*Acidobacteria*	0.01	1.88
6	*Koribacter* sp. (GQ339162)	Iron(II)-rich seep	99.3	*Acidobacteria*	0.54	1.41
7	*Koribacter* sp. (EU150193)	Soil from spruce fir forest	99.8	*Acidobacteria*	1.24	0.40
8	*Oryzihumus* sp. (4P001838)‡	ND	98.3	*Actinobacteria*	0.51	1.19
9	*Granulicella* sp. (FJ466102)	Volcanic deposit	99.8	*Acidobacteria*	0.97	1.13
10	EU861899_s in *Solirubrobacterales* (EU861899)	Meadow surface soil	100	*Actinobacteria*	0.69	1.10
11	*Gallionella* sp. (4P002107)‡	ND	99.3	*Betaproteobacteria*	0.43	1.08

a eztaxon-e database (Kim *et al*., 2012; http://eztaxon-e.ezbiocloud.net/).

b Data for detection sources were from NCBI or publications.

c Accession number was from eztaxon-e.

### Association between bacterial community structure and physical and chemical properties of soil

Significant associations were detected between soil bacterial community and physical and chemical properties of soil. In general, pH showed the highest correlation (*r* = 0.392, *P* < 0.001) with bacterial community composition in all soil samples (Table 3, Fig. 2b). *Acidobacteria*, *Alphaproteobacteria* and *Gammaproteobacteria* decreased with increasing soil pH, whereas *Chloroflexi*, *Betaproteobacteria*, *Bacteroidetes,* and *Deltaproteobacteria* increased with increasing soil pH (Fig. 1). Similar results were observed in the upper and lower-layer soils. At the class or order level, the bacterial community structure changed along the pH gradient, and some taxa showed opposite responses to pH compared with that at the phylum level (Fig. S1). For example, uncultured iii1-8 and *Acidobacteria*-6 of the *Acidobacteria*, *Rhizobiales* of the *Alphaproteobacteria*, and *Legionellales* of the *Gammaproteobacteria* increased with increasing soil pH, whereas *Ktedonobacteria* of the *Chloroflexi* decreased with increasing soil pH.

**Table 3:** The significant correlations between physicochemical properties of soil and bacterial communities

Soil physical and chemical properties	All soil samples (*n* = 70)		Upper-layer (*n* = 36)		Lower-layer (*n* = 34)	
		
	*r*	*P*	*r*	*P*	*r*	*P*
pH	0.392	**0.001**	0.393	**0.001**	0.395	**0.001**
C/N	0.112	**0.021**	0.148	0.054	0.213	**0.025**
MC	0.212	**0.001**	0.122	0.094	0.257	**0.005**
TC	0.196	**0.003**	0.168	0.062	0.116	0.137
TN	0.171	**0.005**	0.323	**0.002**	0.137	0.077
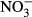	0.001	0.375	0.020	0.392	−0.039	0.656
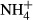	0.035	0.191	0.167	**0.016**	−0.044	0.691

The Spearman's rank correlations (*r*) and significance (*P*) were determined by Mantel tests.

C/N, a ratio of carbon and nitrogen; MC, moisture content; TC, total carbon; TN, total nitrogen.

Significant correlation (*P* < 0.05) values are in bold.

Besides soil pH, C/N ratio (*r* = 0.112, *P* < 0.05), MC (*r* = 0.212, *P* < 0.001), TC (*r* = 0.196, *P* < 0.005), and TN (*r* = 0.171, *P* < 0.005) showed significant correlation with the overall soil bacterial community composition (Table 3). However, different soil properties were associated with the bacterial community structure in the two soil layers; in the upper-layer soils, TN (*r* = 0.323, *P* < 0.005) and 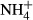 (*r* = 0.167, *P* < 0.05) were significantly associated with the community composition, whereas C/N ratio (*r* = 0.213, *P* < 0.05) and MC (*r* = 0.257, *P* < 0.005) were significantly associated with the community composition in the lower-layer soils (Table 3).

**Fig. 4:**
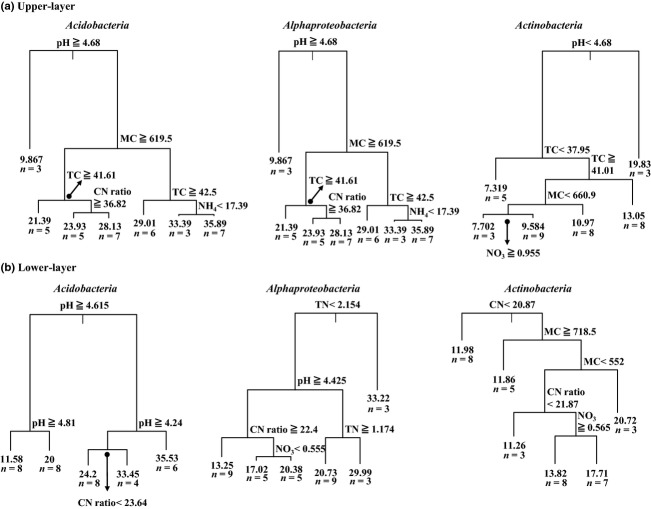
cart analysis to describe the main properties for the dominant phyla *Acidobacteria*, *Alphaproteobacteria*, and *Actinobacteria* in the upper (a) and the lower-layer soil (b) samples.

To identify the most influential soil properties, correlations between physical and chemical properties of soil and the dominant groups were determined using cart analysis (Fig. 4). The soil characteristics affecting each dominant group differed between the soil depths. Soil pH was the best predictor for the presence of *Acidobacteria* in both soil layers. The presence of *Alphaproteobacteria* was related to pH in the upper-layer soils and was related to TN in the lower-layer soils. *Actinobacteria* was related to pH in the upper-layer soils and was related to C/N ratio in the lower-layer soils (Fig. 4).

## Discussion

We investigated the bacterial community structure and its relationships with soil properties in subarctic tundra soils in Council, Alaska. The results showed that the bacterial community in the moist acidic tussock tundra soil was very diverse and that soil depth and pH were the properties that were most influential in predicting the bacterial community structure.

The similarity of the bacterial community was more different through the vertical depth (10 cm) than across the horizontal layers (> 25 m). Soil depth is one of the major parameters influencing microbial community. Waldrop & Harden (2008) showed that microbial biomass and activity significantly decreased at the surface following wildfire; however, the effects of wildfire on those changes were not obvious at 20 cm depth. Furthermore, while long-term warming significantly decreased the evenness of bacterial communities at the surface organic layer soils, the effect of warming was relatively minor in the mineral layer (Deslippe *et al*., 2012). Previous studies indicated that this vertical variation was due to numerous soil properties that change with soil depth, such as pH, nutrient and water availability, plants, soil structure, oxygen, and temperature (Fierer *et al*., 2003; Ström *et al*., 2003; Kobabe *et al*., 2004; Hansel *et al*., 2008). In this study, we also found that there were significant differences in soil pH, MC, TC concentration, and C/N ratio between the two soil depths (*P* < 0.05, Table 1), whereas no obvious trends in soil properties were observed among soils obtained at the same depth.

Among the three major groups, the relative abundances of *Alphaproteobacteria* decreased with depth, although those of *Acidobacteria* and *Actinobacteria* were similar in both soil layers (Fig. S2). This observation corresponded with other observations of bacterial community composition changes with soil depth (Eilers *et al*., 2012; Frank-Fahle *et al*., 2014). *Alphaproteobacteria* prefer nutrient-rich environments (Nemergut *et al*., 2010; Thomson *et al*., 2010; Goldfarb *et al*., 2011). Moreover, Fierer *et al*. (2012) showed the increase in the relative abundance for *Alphaproteobacteria* with additional N input. The decomposition degree of plant and moss differed at different soil depths. The lower C/N ratio in the lower-layer soil reflected more decomposition in the lower-layer than in the upper-layer (Table 1). Therefore, labile materials that provide nutrients for bacteria might be more abundant in the upper-layer soils. In addition, the concentrations of TC, TN, and 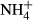 were higher in the upper-layer soils than in the lower-layer soils (Table S1). The environment in the upper-layer would favor *Alphaproteobacteria* propagation.

Soil pH was significantly correlated with bacterial community structure in both layers (Table 3; Fig. 2b). Specifically, *Acidobacteria*, *Alphaproteobacteria*, and *Gammaproteobacteria* decreased with increasing soil pH, whereas *Betaproteobacteria* and *Chloroflexi* increased with increasing pH (Fig. 1). Even when we excluded the dominant groups (*Alphaproteobacteria*, *Acidobacteria*, and *Actinobacteria*) from the statistical analyses, the minor groups also showed significant correlations with soil pH (*P* < 0.001) in both upper- and lower-layer soils (Table S3). These results corresponded with other studies of Arctic and subarctic soils (Männistö *et al*., 2007; Chu *et al*., 2010). Männistö *et al*. (2007) showed that the soil pH of parent materials had greater influence on bacterial community structure than changes in soil temperature in the subarctic region. Chu *et al*. (2010) compared bacterial community structure on a global scale, and concluded that bacterial community composition in arctic soil was strongly influenced by local environmental factors associated with soil acidity than by other factors. Moreover, soil pH is known as a strong driver shaping bacterial community structure in various soil ecosystems, including a wide range of soils in North and South America and agricultural soil in Scotland (Fierer & Jackson, 2006; Lauber *et al*., 2009; Bartram *et al*., 2014).

At lower taxonomic levels, some taxa showed responses to pH opposite to that observed at the phylum level. For example, *Acidobacteria*-6 and iii1-8 (*Acidobacteria*), and *Legionellales* (*Gammaproteobacteria*) increased with increasing soil pH, whereas *Ktedonobacteria* (*Chloroflexi*) decreased with increasing soil pH (Fig. S1). Our results agreed with other studies to show the relationship between soil pH and the lower taxonomic levels of bacteria (Jones *et al*., 2009; Bartram *et al*., 2014). Although these taxa were not the dominant groups, it is noteworthy to examine the different responses of bacterial groups at the lower taxonomic levels because not all members of the same phylum behaved in the same way.

The dominant bacterial phyla in the moist acidic tussock tundra soil in this study were *Acidobacteria*, *Alphaproteobacteria*, and *Actinobacteria*. It is consistent with other studies of various subarctic and Arctic soils, such as tundra soil from Nunavut, the Toolik Lake area, and spanning the Arctic region (Neufeld & Mohn, 2005; Wallenstein *et al*., 2007; Campbell *et al*., 2010; Chu *et al*., 2010; Nemergut *et al*., 2010; Schütte *et al*., 2010). Bacterial composition, including minor groups, was also similar to that of other Arctic soils (Zhou *et al*., 1997; Neufeld & Mohn, 2005; Wallenstein *et al*., 2007; Campbell *et al*., 2010; Chu *et al*., 2010; Nemergut *et al*., 2010; Schütte *et al*., 2010). Moreover, the three dominant phyla are also dominant in soils from nonpolar areas (Fierer & Jackson, 2006; Will *et al*., 2010; Li *et al*., 2012; Shen *et al*., 2013). According to a review by Janssen (2006), these phyla are ubiquitous, and they are the most abundant phyla in soils from various ecosystems. However, soil bacterial community in Hess Creek, Alaska showed different community structure as *Actinobacteria*, *Proteobacteria*, and *Chloroflexi* were dominant (Mackelprang *et al*., 2011). Because *Chloroflexi* increased with increasing soil pH, the abundant *Chloroflexi* in Hess Creek can be attributed to higher soil pH range of 6.43–6.52 in this area (Mackelprang *et al*., 2011).

The higher resolution of pyrosequencing data allowed us to look into the information on the potential ecological roles of bacteria in the subarctic tundra soil. At the species level, OTU_1 (99.5% sequence similarity with *Afipia broomeae*) and OTU_2 (98.5% sequence similarity with *Pseudolabrys* sp.) were predominant in this study (Table 2). They belonged to *Rhizobiales* (*Alphaproteobacteria*) which are known to fix nitrogen as plant root symbionts. The genus *Steroidobacter* of *Gammaproteobacteria* can reduce nitrate to dinitrogen monoxide and further to dinitrogen (Fahrbach *et al*., 2008). *Gallionella* of *Betaproteobacteria* is characterized by its oxidation of Fe (II) (Hedrich *et al*., 2011). Methane-consuming bacteria which belong to the family *Methylocystaceae* (*Alphaproteobacteria*) were detected in this study as well (Fig. S2). These results provide some information on the ecological roles of bacteria in tundra soil.

The distance-decay relationships showed that bacterial community similarity decreased with increasing sampling distance in lower-layer soils, whereas no significant relationship was detected in the upper-layer soils (Fig 4). Decreasing bacterial community similarity with distance can be explained by increasing differences in environmental properties (Nekola & White, 1999). Although it may not be direct evidence of dissimilarity in environmental properties across distance, we observed greater variation in soil properties (TC and TN concentrations and MC) in the lower-layer soils than in the upper-layer soils (Table 1). There is currently no consensus on the biogeographical patterns of bacterial communities. Several studies showed that bacterial community similarity decreased with increasing sampling distance. Monroy *et al*. (2012) reported that bacterial community composition changed with geographic distance (1–200 km range); however, they could not explain this relationship using measured soil properties. Stres *et al*. (2013) also observed an increasing pattern of bacterial community dissimilarity with distance in topographically complex high-altitude slopes in the Himalaya (1–1200 m range). However, other studies reported no biogeographical pattern in microbial community similarity, including bacteria, on either a local or a global scale (Ritz *et al*., 2004; Chu *et al*., 2010; Queloz *et al*., 2011). We also found contrasting results for the relationship between bacterial community similarity and sampling distance between the two depths; no differences were observed in the upper layer, whereas increasing dissimilarity with distance was observed in the lower layer. Therefore, additional research is needed to determine the relationships between geological distance and bacterial community structure.

In summary, soil depth and pH were the most influential soil properties to determine the bacterial community structure in subarctic tundra soil in Council, Alaska. Although various plants covered the top soil, the bacterial communities were relatively similar across the horizontal layers compared with the communities through the vertical depth. Bacterial communities were more significantly correlated with soil pH than the other measured soil properties. These results indicated that the bacterial communities in this study differed at the two different soil depths and were relatively stable against various soil properties except soil pH. Moreover, we found that certain phylogenetic groups at lower taxonomic levels showed a different response to pH from that at the phylum level. This indicated the necessity of analyzing bacterial communities at lower taxonomic levels such as species, which actually perform various functions in the environment. More metagenomic and transcriptomic studies are needed to understand the community structure of other soil microorganisms such as archaea and fungi, and the ecological functions of microorganisms in tundra soil.

## Supporting Information

**Table S1.** The physical and chemical properties of the subarctic soil samples. **Table S2.** Summary statistics of pyrosequencing 16S rRNA gene sequences of soil samples. **Table S3.** The significant correlations between physicochemical properties of soil and bacterial minor groups*. **Table S4.** The list of barcode sequences in this study. **Fig. S1.** Bacterial community structures at class level of *Acidobacteria* (a, b) and *Chloroflexi* (c, d), and at order level of *Alphaproteobacteira* (e, f) and *Gammaproteobacteria* (g, h) with soil pH category. **Fig. S2.** Hierarchical classifications of 16S rRNA gene sequences in the eight main bacterial communities.
