# Supplementary material for: Bacterial community structure and soil properties of a subarctic tundra soil in Council, Alaska
Source: FEMS Microbiol Ecol. 2014 Aug 4;89(2):465–75. doi: 10.1111/1574-6941.12362 (PMC4143960; doi:10.1111/1574-6941.12362)
Supplement: Supplementary file 1 [file fem0089-0465-SD1.docx]

Supporting Information

Additional Supporting Information may be found in the online version of this article:

Table S1. The physical and chemical properties of the subarctic soil samples

Table S2. Summary statistics of pyrosequencing 16S rRNA gene sequences of soil samples

Table S3. The significant correlations between physicochemical properties of soil and bacterial minor groups^*^. The Spearman's rank correlations (*r*) and significance (*p*) were determined by Mantel tests. C/N, ratio of carbon and nitrogen; MC, moisture content; TC, total carbon; TN, total nitrogen

Table S4. The list of barcode sequences in this study.

Fig. S1. Bacterial community structures at class level of *Acidobacteria* (A, B) and *Chloroflexi* (C, D), and at order level of *Alphaproteobacteira* (E, F) and *Gammaproteobacteria* (G, H) with soil pH category.

Fig. S2. Hierarchical classifications of 16S rRNA gene sequences in the eight main bacterial communities. The rings show the soil bacterial community composition at different taxonomic levels, the innermost ring indicates the composition at the phylum level, and the other rings show the composition at the class, order, and family. 1, *Proteobacteria*; 2, *Acidobacteria*; 3, *Actinobacteria*; 4, *Chloroflexi*; 5, *Bacteroidetes*; 6, AD3; 7, *Verrucomicrobia*; 8, *Planctomycetes*; 9, *Alphaproteobacteria*; 10, *Betaproteobacteria*; 11, *Deltaproteobacteria*; 12, *Gammaproteobacteria.*

Supplementary data

Table S1. The physical and chemical properties of the subarctic soil samples

| Sampling sites | pH | | TC (%) | | TN (%) | | C/N | | MC (%) | | NO_3_^-^ | | NH_4_^+^ | |
| --- | --- | --- | --- | --- | --- | --- | --- | --- | --- | --- | --- | --- | --- | --- |
|  |  |  |  |  |  |  |  |  |  |  | (μg N g^-1^ soil) | | (μg N g^-1^ soil) | |
|  | Upper | Lower | Upper | Lower | Upper | Lower | Upper | Lower | Upper | Lower | Upper | Lower | Upper | Lower |
| 1 | 4.04 | 4.08 | 39.45 | 42.91 | 1.00 | 1.52 | 39.56 | 28.20 | 667.6 | 752.5 | 0.58 | 0.49 | 23.90 | 16.74 |
| 2 | 4.00 | 4.33 | 41.92 | 24.84 | 1.60 | 1.13 | 26.18 | 22.06 | 585.4 | 187.8 | 0.53 | 0.21 | 17.71 | 20.03 |
| 3 | 4.55 | 4.50 | 42.76 | 45.38 | 2.22 | 1.72 | 19.31 | 26.41 | 506.8 | 405.9 | 1.09 | 0.69 | 93.08 | 30.88 |
| 4 | 4.63 | - | 40.54 | - | 1.89 | - | 21.46 | - | 746.5 | - | 0.55 | - | 80.26 | - |
| 5 | 4.51 | 4.66 | 36.56 | 42.32 | 1.16 | 1.96 | 31.54 | 21.58 | 960.2 | 789.5 | 0.59 | 0.42 | 21.01 | 35.62 |
| 6 | 4.65 | 4.64 | 40.05 | 48.55 | 1.54 | 1.99 | 25.98 | 24.45 | 618.3 | 484.2 | 0.27 | 0.28 | 45.09 | 26.18 |
| 7 | 4.29 | 4.41 | 43.64 | 47.21 | 1.73 | 2.13 | 25.29 | 22.22 | 609.4 | 547.2 | 0.48 | 0.55 | 28.46 | 91.55 |
| 8 | 4.23 | 4.59 | 40.52 | 41.85 | 1.04 | 1.75 | 38.89 | 23.95 | 871.2 | 264.9 | 0.42 | 0.34 | 12.18 | 30.88 |
| 9 | 4.14 | 3.96 | 38.08 | 48.33 | 1.32 | 1.85 | 28.89 | 26.18 | 536.9 | 509.4 | 1.15 | 1.10 | 63.81 | 37.69 |
| 10 | 4.10 | 4.34 | 40.61 | 44.37 | 1.67 | 1.91 | 24.27 | 23.27 | 368.5 | 382.8 | 0.39 | 1.28 | 53.67 | 36.44 |
| 11 | 4.66 | 4.72 | 37.18 | 43.18 | 1.67 | 2.14 | 22.32 | 20.22 | 464.5 | 441.8 | 0.41 | 0.50 | 17.08 | 12.96 |
| 12 | 4.38 | 4.69 | 41.78 | 9.38 | 1.30 | 0.36 | 32.14 | 26.05 | 984.7 | 143.1 | 0.55 | 0.39 | 33.80 | 11.10 |
| 13 | 4.10 | 4.43 | 41.49 | 45.52 | 1.28 | 2.17 | 32.31 | 21.02 | 760.9 | 556.8 | 0.52 | 0.74 | 20.55 | 21.49 |
| 14 | 4.14 | 4.67 | 42.40 | 40.21 | 1.71 | 1.62 | 24.87 | 24.84 | 302.6 | 292.0 | 0.71 | 0.55 | 55.50 | 45.18 |
| 15 | 4.70 | 4.93 | 41.66 | 17.71 | 1.80 | 0.79 | 23.20 | 22.47 | 518.5 | 311.7 | 1.16 | 0.58 | 43.16 | 28.81 |
| 16 | 4.53 | 4.85 | 43.12 | 15.44 | 1.98 | 0.75 | 21.74 | 20.53 | 439.8 | 123.6 | 1.15 | 0.39 | 15.58 | 19.47 |
| 17 | 5.02 | 4.83 | 2.10 | 1.85 | 0.09 | 0.08 | 22.57 | 22.51 | 53.4 | 32.1 | 0.38 | 0.36 | 32.99 | 22.32 |
| 18 | 4.04 | 4.13 | 43.84 | 45.85 | 1.79 | 2.14 | 24.53 | 21.44 | 680.3 | 261.0 | 1.01 | 0.49 | 17.11 | 26.65 |
| 19 | 4.07 | 4.34 | 42.79 | 48.07 | 1.86 | 2.32 | 22.97 | 20.73 | 451.0 | 366.0 | 1.00 | 0.52 | 12.73 | 27.39 |
| 20 | 4.50 | 4.82 | 37.36 | 39.91 | 1.66 | 1.94 | 22.51 | 20.56 | 533.1 | 477.7 | 3.19 | 3.29 | 12.65 | 30.01 |
| 21 | 4.48 | 4.80 | 41.08 | 26.44 | 1.78 | 1.25 | 23.12 | 21.12 | 644.6 | 395.2 | 0.90 | 0.77 | 42.24 | 46.15 |
| 22 | 4.47 | 4.44 | 41.33 | 44.35 | 1.27 | 1.84 | 32.59 | 24.12 | 1058.9 | 767.0 | 0.60 | 0.80 | 36.13 | 22.27 |
| 23 | 4.03 | 4.19 | 41.73 | 37.37 | 1.23 | 1.34 | 33.90 | 27.84 | 906.0 | 605.1 | 0.58 | 0.85 | 25.22 | 19.44 |
| 24 | 3.90 | 4.19 | 40.94 | 41.82 | 1.01 | 1.40 | 40.61 | 29.89 | 906.1 | 684.6 | 0.82 | 1.04 | 10.62 | 16.20 |
| 25 | 4.15 | 4.51 | 37.82 | 46.01 | 1.13 | 2.38 | 33.50 | 19.31 | 484.0 | 365.0 | 0.82 | 0.83 | 8.62 | 12.27 |
| 26 | 4.65 | 4.82 | 41.87 | 32.48 | 1.96 | 1.45 | 21.33 | 22.34 | 677.2 | 199.3 | 0.93 | 0.69 | 13.07 | 10.27 |
| 27 | 4.57 | 4.64 | 43.02 | 41.92 | 2.10 | 2.14 | 20.54 | 19.57 | 777.0 | 632.9 | 0.83 | 1.05 | 72.65 | 69.96 |
| 28 | 4.28 | 4.29 | 41.46 | 45.32 | 1.42 | 1.97 | 29.14 | 23.03 | 620.6 | 407.6 | 0.79 | 0.77 | 15.40 | 11.92 |
| 29 | 4.14 | 4.33 | 40.78 | 38.99 | 0.97 | 1.22 | 42.22 | 31.91 | 791.2 | 891.1 | 1.00 | 1.27 | 13.99 | 18.65 |
| 30 | 4.03 | 4.33 | 43.25 | 23.87 | 1.58 | 1.02 | 27.46 | 23.33 | 423.4 | 289.4 | 0.74 | 0.87 | 14.66 | 9.79 |
| 31 | 4.62 | 4.96 | 42.60 | 24.69 | 1.89 | 1.22 | 22.59 | 20.31 | 558.2 | 139.4 | 0.84 | 0.47 | 21.27 | 16.60 |
| 32 | 4.81 | 5.01 | 43.87 | 33.48 | 2.23 | 1.46 | 19.64 | 22.88 | 590.2 | 339.1 | 0.96 | 0.64 | 55.57 | 25.28 |
| 33 | 4.58 | 4.85 | 41.45 | 15.80 | 1.90 | 0.87 | 21.79 | 18.26 | 351.2 | 111.2 | 0.72 | 0.56 | 48.95 | 29.78 |
| 34 | 4.61 | 4.74 | 35.88 | 37.13 | 1.34 | 1.71 | 26.88 | 21.68 | 472.3 | 546.4 | 0.57 | 0.55 | 62.47 | 61.47 |
| 35 | 4.07 | - | 41.35 | - | 1.19 | - | 34.75 | - | 626.3 | - | 0.91 | - | 15.83 | - |
| 36 | 3.95 | 4.12 | 41.44 | 39.57 | 0.63 | 0.70 | 66.30 | 56.93 | 1070.3 | 1201.6 | 0.79 | 1.14 | 13.99 | 46.69 |

TC, total carbon; TN, total nitrogen; C/N, a ratio of carbon to nitrogen; MC, moisture content.

-, no data

Table S2. Summary statistics of pyrosequencing 16S rRNA gene sequences of soil samples

| Sampling sites | No. of bacterial reads | |  | No. of observed OTUs^a^ | |  | Alpha diversity measures^*^ | | | | | | | | | | | | | | | | | | | | | | |
| --- | --- | --- | --- | --- | --- | --- | --- | --- | --- | --- | --- | --- | --- | --- | --- | --- | --- | --- | --- | --- | --- | --- | --- | --- | --- | --- | --- | --- | --- |
|  |  |  |  |  |  |  | OTUs | | | |  | Chao1 | | | |  | Shannon (*H*') | | | |  | Simpson | | | |  | Estimated coverage (%) | | |
|  | Upper | Lower |  | Upper | Lower |  | Upper | Lower |  | Upper | | | Lower |  | Upper | | | Lower |  | Upper | | | Lower |  | Upper | | | Lower |  |
| 1 | 1870 | 1590 |  | 474 | 397 |  | 260 | 211 |  | 611 | | | 517 |  | 6.87 | | | 6.89 |  | 0.97 | | | 0.98 |  | 86.1 | | | 86.7 |  |
| 2 | 1944 | 1573 |  | 368 | 275 |  | 201 | 134 |  | 502 | | | 372 |  | 6.32 | | | 5.98 |  | 0.97 | | | 0.96 |  | 89.7 | | | 91.5 |  |
| 3 | 1062 | 923 |  | 231 | 213 |  | 118 | 113 |  | 352 | | | 352 |  | 6.11 | | | 6.27 |  | 0.96 | | | 0.97 |  | 88.9 | | | 87.8 |  |
| 4 | 1353 | - |  | 495 | - |  | 296 | - |  | 795 | | | - |  | 7.58 | | | - |  | 0.99 | | | - |  | 78.1 | | | - |  |
| 5 | 1298 | 908 |  | 429 | 285 |  | 247 | 175 |  | 681 | | | 599 |  | 7.25 | | | 6.97 |  | 0.98 | | | 0.98 |  | 81.0 | | | 80.7 |  |
| 6 | 988 | 984 |  | 362 | 307 |  | 222 | 183 |  | 661 | | | 562 |  | 7.16 | | | 6.94 |  | 0.98 | | | 0.98 |  | 77.5 | | | 81.4 |  |
| 7 | 1372 | 1360 |  | 375 | 204 |  | 202 | 111 |  | 529 | | | 289 |  | 6.86 | | | 5.60 |  | 0.98 | | | 0.96 |  | 85.3 | | | 91.8 |  |
| 8 | 1225 | 1706 |  | 409 | 304 |  | 260 | 161 |  | 679 | | | 439 |  | 7.13 | | | 6.17 |  | 0.98 | | | 0.97 |  | 78.8 | | | 90.6 |  |
| 9 | 1448 | 718 |  | 463 | 178 |  | 273 | 107 |  | 741 | | | 426 |  | 7.18 | | | 6.23 |  | 0.98 | | | 0.97 |  | 81.1 | | | 85.1 |  |
| 10 | 1580 | 1153 |  | 433 | 339 |  | 249 | 208 |  | 620 | | | 588 |  | 6.87 | | | 6.62 |  | 0.97 | | | 0.97 |  | 84.2 | | | 82.0 |  |
| 11 | 1693 | 876 |  | 497 | 260 |  | 296 | 149 |  | 678 | | | 467 |  | 7.24 | | | 6.70 |  | 0.99 | | | 0.98 |  | 82.5 | | | 83.0 |  |
| 12 | 1373 | 1397 |  | 388 | 274 |  | 224 | 142 |  | 596 | | | 395 |  | 6.83 | | | 6.16 |  | 0.98 | | | 0.97 |  | 83.7 | | | 89.8 |  |
| 13 | 1765 | 1262 |  | 379 | 229 |  | 213 | 127 |  | 503 | | | 349 |  | 6.52 | | | 5.57 |  | 0.98 | | | 0.95 |  | 87.9 | | | 89.9 |  |
| 14 | 1687 | 1584 |  | 356 | 333 |  | 186 | 195 |  | 447 | | | 463 |  | 6.63 | | | 6.20 |  | 0.98 | | | 0.96 |  | 89.0 | | | 87.7 |  |
| 15 | 1694 | 1574 |  | 334 | 269 |  | 173 | 139 |  | 442 | | | 383 |  | 6.39 | | | 6.06 |  | 0.97 | | | 0.97 |  | 89.8 | | | 91.2 |  |
| 16 | 1266 | 1346 |  | 470 | 388 |  | 283 | 210 |  | 742 | | | 541 |  | 7.36 | | | 6.87 |  | 0.98 | | | 0.98 |  | 77.6 | | | 84.4 |  |
| 17 | 1416 | 1455 |  | 348 | 319 |  | 181 | 163 |  | 459 | | | 429 |  | 6.80 | | | 6.52 |  | 0.98 | | | 0.97 |  | 87.2 | | | 88.8 |  |
| 18 | 1635 | 1090 |  | 450 | 154 |  | 256 | 87 |  | 658 | | | 258 |  | 7.10 | | | 4.80 |  | 0.99 | | | 0.91 |  | 84.3 | | | 92.0 |  |
| 19 | 1510 | 927 |  | 381 | 246 |  | 205 | 138 |  | 520 | | | 416 |  | 6.75 | | | 6.38 |  | 0.97 | | | 0.97 |  | 86.4 | | | 85.1 |  |
| 20 | 1432 | 1389 |  | 452 | 286 |  | 250 | 164 |  | 671 | | | 420 |  | 7.16 | | | 6.22 |  | 0.98 | | | 0.97 |  | 82.5 | | | 88.2 |  |
| 21 | 1351 | 1222 |  | 339 | 252 |  | 196 | 142 |  | 507 | | | 383 |  | 6.71 | | | 6.07 |  | 0.98 | | | 0.96 |  | 85.5 | | | 88.4 |  |
| 22 | 1341 | 1602 |  | 410 | 201 |  | 237 | 88 |  | 623 | | | 240 |  | 7.02 | | | 5.56 |  | 0.98 | | | 0.96 |  | 82.3 | | | 94.5 |  |
| 23 | 1297 | 1493 |  | 329 | 275 |  | 180 | 128 |  | 485 | | | 365 |  | 6.81 | | | 6.26 |  | 0.98 | | | 0.97 |  | 86.1 | | | 91.4 |  |
| 24 | 1303 | 1838 |  | 371 | 175 |  | 220 | 73 |  | 603 | | | 175 |  | 7.00 | | | 5.16 |  | 0.98 | | | 0.93 |  | 83.1 | | | 96.0 |  |
| 25 | 1706 | 1445 |  | 515 | 393 |  | 311 | 227 |  | 783 | | | 562 |  | 7.24 | | | 6.53 |  | 0.99 | | | 0.96 |  | 81.8 | | | 84.3 |  |
| 26 | 1255 | 1080 |  | 422 | 261 |  | 253 | 141 |  | 674 | | | 417 |  | 7.29 | | | 6.32 |  | 0.99 | | | 0.97 |  | 79.8 | | | 86.9 |  |
| 27 | 1182 | 999 |  | 427 | 325 |  | 251 | 182 |  | 647 | | | 536 |  | 7.65 | | | 7.18 |  | 0.99 | | | 0.99 |  | 78.8 | | | 81.8 |  |
| 28 | 842 | 1593 |  | 310 | 312 |  | 199 | 168 |  | 649 | | | 470 |  | 6.95 | | | 6.48 |  | 0.98 | | | 0.98 |  | 76.4 | | | 89.5 |  |
| 29 | 1314 | 1388 |  | 306 | 275 |  | 154 | 146 |  | 416 | | | 363 |  | 6.49 | | | 6.17 |  | 0.97 | | | 0.97 |  | 88.3 | | | 89.5 |  |
| 30 | 973 | 1388 |  | 214 | 227 |  | 107 | 106 |  | 327 | | | 286 |  | 6.22 | | | 6.06 |  | 0.97 | | | 0.97 |  | 89.0 | | | 92.4 |  |
| 31 | 1261 | 1036 |  | 345 | 257 |  | 192 | 133 |  | 499 | | | 392 |  | 6.80 | | | 6.46 |  | 0.98 | | | 0.98 |  | 84.8 | | | 87.2 |  |
| 32 | 1098 | 1279 |  | 341 | 318 |  | 198 | 177 |  | 582 | | | 467 |  | 6.97 | | | 6.65 |  | 0.98 | | | 0.98 |  | 82.0 | | | 86.2 |  |
| 33 | 1223 | 1278 |  | 373 | 398 |  | 212 | 220 |  | 592 | | | 578 |  | 7.00 | | | 7.22 |  | 0.98 | | | 0.98 |  | 82.7 | | | 82.8 |  |
| 34 | 777 | 745 |  | 258 | 234 |  | 160 | 129 |  | 534 | | | 442 |  | 6.66 | | | 6.72 |  | 0.98 | | | 0.98 |  | 79.4 | | | 82.7 |  |
| 35 | 1139 | - |  | 294 | - |  | 156 | - |  | 417 | | | - |  | 6.74 | | | - |  | 0.98 | | | - |  | 86.3 | | | - |  |
| 36 | 857 | 1011 |  | 265 | 351 |  | 155 | 213 |  | 483 | | | 649 |  | 6.76 | | | 7.24 |  | 0.98 | | | 0.98 |  | 81.9 | | | 78.9 |  |

-, no data

^a^ The number of OTUs was generated at the 97% sequence similarity cutoff.

^*^ Diversity indices represent the randomly selected subsets (n=700) for each sample.

Table S3. The significant correlations between physicochemical properties of soil and bacterial minor groups^*^. The Spearman's rank correlations (*r*) and significance (*p*) were determined by Mantel tests. C/N, ratio of carbon and nitrogen; MC, moisture content; TC, total carbon; TN, total nitrogen

| Soil physical and chemical properties | All soil samples (n=70) | |  | Upper layer (n=36) | |  | Lower layer (n=34) | |
| --- | --- | --- | --- | --- | --- | --- | --- | --- |
|  | *r* | *p* |  | *r* | *p* |  | *r* | *p* |
| pH | 0.422 | 0.001 |  | 0.427 | 0.001 |  | 0.350 | 0.001 |
| C/N | 0.032 | 0.251 |  | -0.012 | 0.496 |  | 0.178 | 0.185 |
| MC | 0.081 | 0.04 |  | -0.011 | 0.515 |  | 0.140 | 0.043 |
| TC | 0.212 | 0.002 |  | 0.147 | 0.091 |  | 0.157 | 0.040 |
| TN | 0.140 | 0.003 |  | 0.300 | 0.003 |  | 0.126 | 0.047 |
| NO_3_ | 0.002 | 0.466 |  | 0.049 | 0.285 |  | 0.014 | 0.392 |
| NH_4_ | 0.062 | 0.111 |  | 0.312 | 0.004 |  | -0.045 | 0.693 |

^*^Bacterial minor groups except major groups (*Alphaproteobacteria*, *Acidobacteria*, and *Actinobacteria*).

Table S4. The list of barcode sequences in this study.

| #SampleID | BarcodeSequence |  | #SampleID | BarcodeSequence |
| --- | --- | --- | --- | --- |
| L01 | ACAGACAG |  | U01 | ACACACTG |
| L02 | ACAGCAGA |  | U02 | ACAGAGAC |
| L03 | ACGACATC |  | U03 | ACAGTCAC |
| - | - |  | U04 | ACGTCTAG |
| L05 | ACTCTCAC |  | U05 | ACTCAGAC |
| L06 | ACTGCAGT |  | U06 | ACTGACTG |
| L07 | AGACTCAC |  | U07 | AGACACTC |
| L08 | AGCAGATG |  | U08 | AGAGTGTG |
| L09 | AGTCACAC |  | U09 | GTGAGACA |
| L10 | AGTCGTGT |  | U10 | AGTCAGAG |
| L11 | AGTGTCAC |  | U11 | AGTGAGAC |
| L12 | ATCGATGC |  | U12 | ATATCGCG |
| L13 | ATGCTAGC |  | U13 | ATCGTAGC |
| L14 | CACAGAGA |  | U14 | CACACAGT |
| L15 | CACTACTC |  | U15 | CACATCTC |
| L16 | CACTGAGT |  | U16 | CACTCAGA |
| L17 | CAGATCAC |  | U17 | CAGAGAGT |
| L18 | CAGTCAGT |  | U18 | CAGTACTG |
| L19 | CATGAGCT |  | U19 | CAGTGAGA |
| L20 | CGCGATAT |  | U20 | CGATCGAT |
| L21 | CTACTCGT |  | U21 | CGTAGCTA |
| L22 | CTCATCTG |  | U22 | CTCAGAGT |
| L23 | CTGACTCT |  | U23 | CTCTAGTC |
| L24 | CTGATCTC |  | U24 | CTGAGTGT |
| L25 | CTGTCACT |  | U25 | CTGTACTC |
| L26 | GACACTGT |  | U26 | CTGTGTCT |
| L27 | GACATCAC |  | U27 | GACAGTCT |
| L28 | GAGACACA |  | U28 | GACTAGTC |
| L29 | GAGTACTC |  | U29 | GAGAGTGT |
| L30 | GATCGTAC |  | U30 | GAGTCTGT |
| L31 | GCATATCG |  | U31 | GATGCATC |
| L32 | GCTACGAT |  | U32 | GCGCATAT |
| L33 | GTAGACGA |  | U33 | GTACGATC |
| L34 | GTCAGAGA |  | U34 | GTCACAGT |
| - | - |  | U35 | GTCATCTC |
| L36 | GTCTCTGT |  | U36 | GTCTACAG |

LinkerPrimerSequence is ACAGAGTTTGATCMTGGCTCAG.

ReversePrimer is GWATTACCGCGGCKGCTG.

Fig. S1.


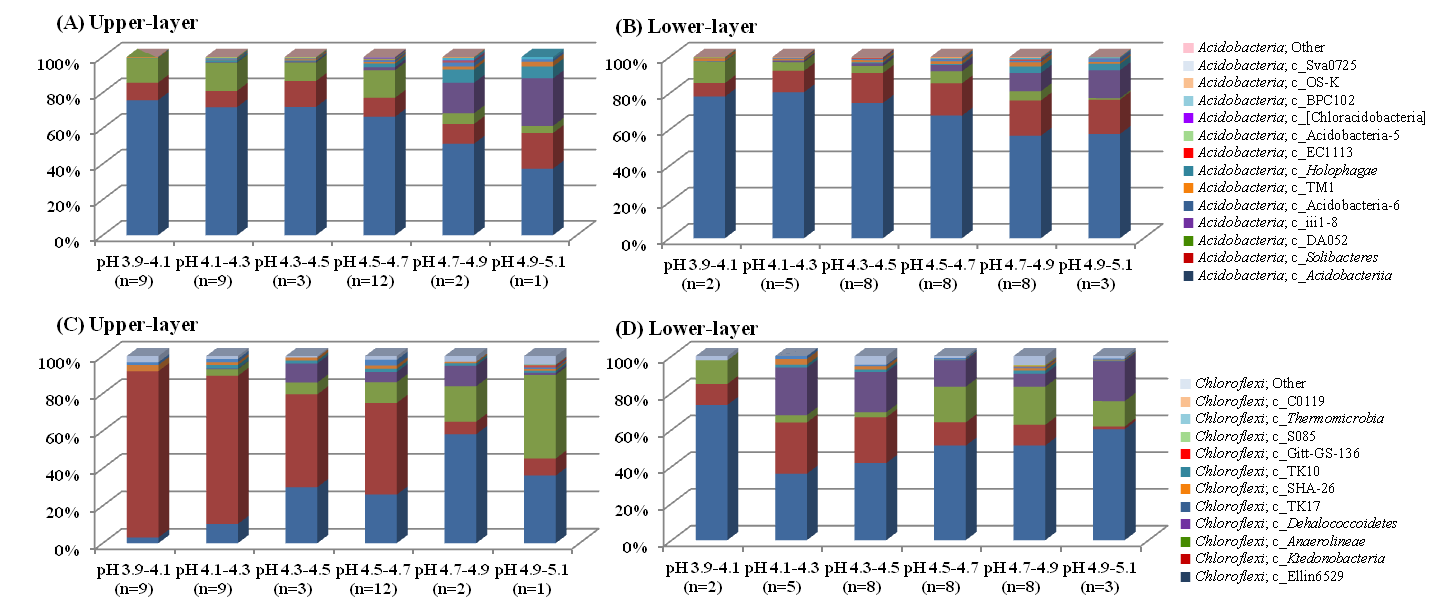


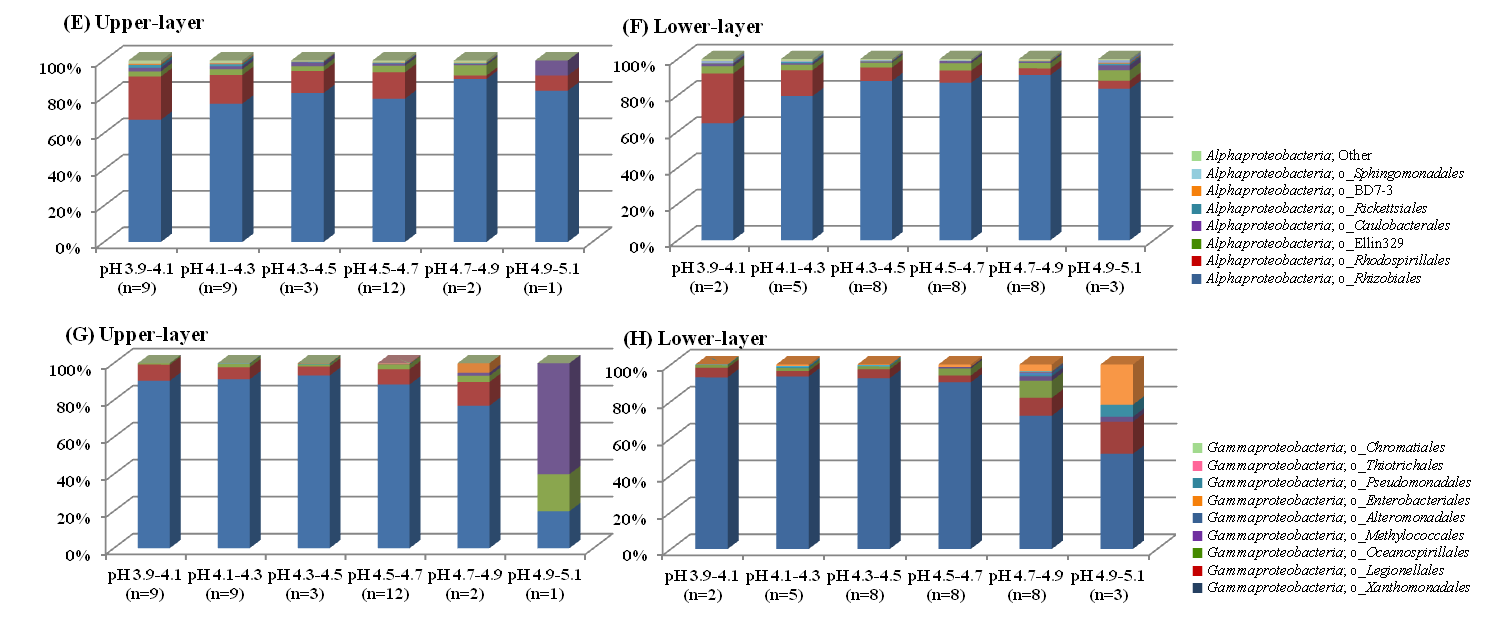


Fig. S1. Bacterial community structures at class level of *Acidobacteria* (A, B) and *Chloroflexi* (C, D), and at order level of *Alphaproteobacteira* (E, F) and *Gammaproteobacteria* (G, H) with soil pH category.

Fig. S2. Hierarchical classifications of 16S rRNA gene sequences in the eight main bacterial communities. The rings show the soil bacterial community composition at different taxonomic levels, the innermost ring indicates the composition at the phylum level, and the other rings show the composition at the class, order, and family. 1, *Proteobacteria*; 2, *Acidobacteria*; 3, *Actinobacteria*; 4, *Chloroflexi*; 5, *Bacteroidetes*; 6, AD3; 7, *Verrucomicrobia*; 8, *Planctomycetes*; 9, *Alphaproteobacteria*; 10, *Betaproteobacteria*; 11, *Deltaproteobacteria*; 12, *Gammaproteobacteria.*
